# Dynamics of Gene Expression Profiling and Identification of High-Risk Patients for Severe COVID-19

**DOI:** 10.3390/biomedicines11051348

**Published:** 2023-05-03

**Authors:** Alexander Rombauts, Marta Bódalo Torruella, Gabriela Abelenda-Alonso, Júlia Perera-Bel, Anna Ferrer-Salvador, Ariadna Acedo-Terrades, Maria Gabarrós-Subirà, Isabel Oriol, Carlota Gudiol, Lara Nonell, Jordi Carratalà

**Affiliations:** 1 Department of Infectious Diseases, Hospital Universitari de Bellvitge-IDIBELL, 08908 Barcelona, Spainisaoriolbermudez@gmail.com (I.O.); cgudiol_ext@iconcologia.net (C.G.);; 2 MARGenomics, Hospital del Mar Medical Research Institute (IMIM), 08003 Barcelona, Spain; mbodalo@imim.es (M.B.T.);; 3 Centre for Genomic Regulation (CRG), The Barcelona Institute of Science and Technology, 08028 Barcelona, Spain; 4 Department of Medicine, Universitat de Barcelona, 08007 Barcelona, Spain; 5 Centro de Investigación Biomédica en Red de Enfermedades Infecciosas (CIBERINFEC), Instituto de Salud Carlos III, 28029 Madrid, Spain

**Keywords:** COVID-19, SARS-CoV-2, transcriptomics, ARDS, gene expression, prognosis

## Abstract

The clinical manifestations of SARS-CoV-2 infection vary widely, from asymptomatic infection to the development of acute respiratory distress syndrome (ARDS) and death. The host response elicited by SARS-CoV-2 plays a key role in determining the clinical outcome. We hypothesized that determining the dynamic whole blood transcriptomic profile of hospitalized adult COVID-19 patients and characterizing the subgroup that develops severe disease and ARDS would broaden our understanding of the heterogeneity in clinical outcomes. We recruited 60 hospitalized patients with RT-PCR-confirmed SARS-CoV-2 infection, among whom 19 developed ARDS. Peripheral blood was collected using PAXGene RNA tubes within 24 h of admission and on day 7. There were 2572 differently expressed genes in patients with ARDS at baseline and 1149 at day 7. We found a dysregulated inflammatory response in COVID-19 ARDS patients, with an increased expression of genes related to pro-inflammatory molecules and neutrophil and macrophage activation at admission, in addition to an immune regulation loss. This led, in turn, to a higher expression of genes related to reactive oxygen species, protein polyubiquitination, and metalloproteinases in the latter stages. Some of the most significant differences in gene expression found between patients with and without ARDS corresponded to long non-coding RNA involved in epigenetic control.

## 1. Introduction

The coronavirus disease 2019 (COVID-19) pandemic, caused by the severe acute respiratory syndrome coronavirus 2 (SARS-CoV-2), the most recent zoonotic coronavirus to cause devastation in humans [1], is a public health problem of historic magnitude. As of March 2023, more than 676 million cases of COVID-19 have been reported globally, with more than 6.8 million deaths [2].

The clinical manifestations of SARS-CoV-2 infections vary broadly, ranging from an asymptomatic state to severe pneumonia, including acute respiratory distress syndrome (ARDS), multisystem organ failure, and eventually death [3,4]. Morbidity and mortality are almost exclusively driven by the development of ARDS. 

An early adaptive immune response with a rapid production of bystander CD8 T cells and plasmablasts with almost no systemic inflammation appears to take place in asymptomatic patients and in those with mild disease [5]. In contrast, progression to severe illness with ARDS has been associated with a proinflammatory immune dysregulation that includes a robust type 2 response [6,7]. Factors such as SARS-CoV-2 viral load [8], immunological imprinting due to previous infections with other coronaviruses [9], autoantibodies against interferon-ω [10], host genetic determinants [11], low levels of type I and III interferons (INF) together with elevated chemokines and high expression of IL-6 [12] may play a role in COVID-19 outcomes. Intriguingly, it remains unclear why some patients develop severe pneumonia with ARDS and others have zero or minimal symptoms.

A genome-wide association found that certain regions, such as 3p21.31, and blood group A, were related to severity [11,12,13]. In addition, several ACE2 polymorphisms [13] and inborn errors of type 1 INF [14] have been correlated with increased severity and susceptibility to COVID-19. 

Regarding transcriptomics, most of the research has analyzed single-cell RNA extracted from peripheral mononuclear cells (PBMC) [15]; only a few studies have been performed on total RNA from whole blood. Some of these whole blood RNA studies focused on identifying a transcriptomic signature differentiating SARS-CoV-2 from influenza [16] and other viral respiratory pathogens [17], while others compared asymptomatic vs. symptomatic patients [6,18]. Investigations evaluating whole blood transcriptomic profiles according to clinical status found that more severe cases showed an upregulation of genes principally related to neutrophil activation [5,17,19,20,21], myeloid cells [20], Il2, Il6, IL8, protein autophagy, protein polyubiquitination [19], TNF-α, and glycolysis [5], while genes related to T-cell activation were under-expressed [16,19,20,22]. As for interferon gene expression, two studies found an enrichment [5,20], while one observed a down-regulation of IFN-γ related genes [19]. 

However, most of the transcriptomic studies of COVID-19 have analyzed a single time point per patient [16,17,19,20,22], thus disregarding the dynamic nature of the disease. In addition, several investigations observed heterogeneity in the transcriptomic profiles among the more severe groups of hospitalized COVID-19 patients [5,21,22].

The main goal of this study was to determine the dynamic transcriptomic profile of adult patients hospitalized for COVID-19 and to characterize the subgroup that developed severe disease and ARDS.

## 2. Materials and Methods

### 2.1. Study Design, Setting, Ethics and Patients

In this prospective study, 60 patients were enrolled at Bellvitge University Hospital. Transcriptomic analyses were performed at the Hospital del Mar Medical Research Institute (IMIM). Adult patients with a positive RT-PCR for SARS-CoV-2 in nasopharyngeal swabs and COVID-19 symptoms requiring hospitalization from 25 March 2020, to 31 July 2020 (during the first wave of the COVID-19 pandemic in Spain) were eligible for recruitment. Patients were enrolled within 24 h of admission. Blood samples were obtained at baseline and on day 7 of hospital admission. Patients were assigned a unique patient identifier (PID), which was applied to the clinical samples and the depersonalized data set. The list correlating the patient’s identity with the PID is securely stored at Bellvitge University Hospital. Patients were prospectively followed-up and seen daily by the investigators. Data on demographic and clinical characteristics, biochemical analysis, treatments, and outcomes were collected in a pseudoanonymized database. The study was approved by the Bellvitge University Hospital Ethics Committee (PR148/20), and written informed consent was obtained for all cases. 

**Figure 1:**
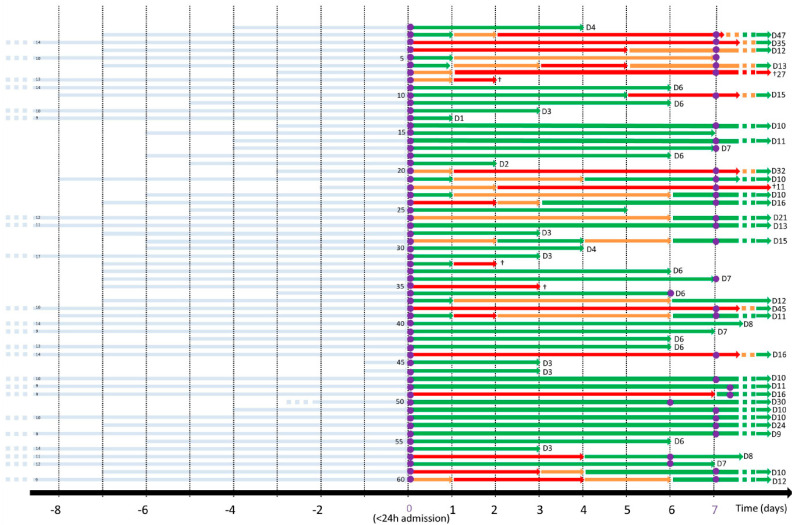
Clinical evolution of each individual patient according to oxygen need and day of sampling. The *x*-axis represents time expressed in days, and the light-blue rows represent symptoms duration prior to hospital admission (day 0). Green represents low oxygen need (oxygen mask up to 8 L/min), orange represents medium oxygen need (between 8 and 15 L/min), and red represents the amplified definition of ARDS (invasive or non-invasive mechanical ventilation, at least 24 h of FiO2 ≥ 70% and high-flow oxygen, ≥15 L/min, delivered by either non-rebreather masks or high-flow nasal oxygen). The purple circles represent peripheral blood sampling using PAXGene RNA tubes. The day of hospital discharge is detailed at the end of each row, and death is represented by the symbol †.

We classified the patients according to their respiratory situation each day over the course of hospitalization (Figure 1). We hypothesized that patients with medium oxygen needs (oxygen mask with oxygen flow between 8 and 15 L/min) could express a transcriptomic profile overlapping ARDS and a more benign clinical evolution. Therefore, to increase the specificity of the transcriptomic profile associated with ARDS, patients who met the amplified definition of ARDS were compared with those with low flow oxygen needs (oxygen masks up to 8 L/min). The transcriptomic profile of patients with low oxygen needs was compared to that of patients with ARDS at baseline. At day 7, the transcriptomic profile of all patients who had developed ARDS at any time was compared with those who had not.

### 2.2. Definitions and Local Guidelines

COVID-19 pneumonia was defined as new or worsening pulmonary infiltrates on a chest x-ray or CT of the lungs with a confirmed positive RT-PCR for SARS-CoV-2. ARDS was defined as acute respiratory failure (PaO2/FiO2 < 300) with bilateral opacities and no acute heart failure. Due to the overburdening of the health-care system and the scarcity of critical care beds during the first COVID-19 wave, we decided to broaden the classical Berlin definition [23] to eliminate the requirement of a positive end-expiratory pressure of at least 5 cmH_2_O. In addition to invasive (IV) or non-invasive mechanical ventilation (NIV), patients who required at least 24 h of FiO2 ≥ 70% and high-flow oxygen (≥15 L/min) delivered by either non-rebreather masks or high-flow nasal oxygen (HFNO) were considered to have ARDS [24]. The case report form and other definitions can be found in the Supplementary Materials section. Corticosteroids and tocilizumab were administered at the attending physician’s discretion. 

### 2.3. RNA Extraction

Peripheral blood was collected using PAXGene RNA tubes from Qiagen. RNA was extracted using a CE-certified PAXGene blood RNA kit at the IMIM’s COVID room with special biosecurity measures (see Supplementary Materials). The quantity and integrity of the samples were assessed with Nanodrop, Qubit, and Bioanalyzer instruments. Samples of sufficient quality were selected for further processing. 

### 2.4. Library Preparation

PAXGene RNA samples were processed using NEBNext Globin rRNA Depletion and NEBNext UltraII DirecRNA LibPrep in order to obtain the libraries. Laboratory parameters (initial input, PCR cycles, and adaptor dilution) were adjusted considering the quantity and quality of the different types of samples. Library profiles were checked using the bioanalyzer instrument and Qubit dsDNA kit to quantify them. Libraries that passed quality control were transferred to the CRG Core Facility. At the CRG, qPCR of the libraries was performed prior to running the flow cell. Samples were sequenced by Illumina HiSeq 2500, resulting in paired 75-nt reads.

### 2.5. RNA-Seq Bioinformatic Processing

Initial quality control was carried out using FastQC (v0.11.5) and FastQ Screen (v0.14.0) and summarized with Multiqc (v1.7). All QC metrics were deemed correct, with a median of 49 million read-pairs per sample. No ribosomal contamination was detected (neither in humans nor in other species). Raw sequencing reads in the fastq files were mapped with STAR version 2.7.1a Gencode release v36 based on the GRCh38.p13 reference genome and the corresponding GTF file. 

### 2.6. Statistical Analysis

SAMtools v1.8 was used to index bam files. The algorithm CollectRnaSeqMetrics from Picard v2.2.4 was used to retrieve alignment metrics. The table of counts was obtained with the featureCounts function in the package subread (v1.6.4). The differential gene expression (DEG) analysis was assessed with voom + limma in the limma package (v3.46.0) using R (v4.0.3). Linear models included the batch as a covariate. Significant mean positive log2-fold changes correspond to upregulation, while negative changes correspond to downregulation. Functional analysis was performed with the clusterProfiler R package (v4.2.2) and the Hallmark collection from the Molecular Signatures Database (MSigDB, v7.5.1). Deconvolution analyses were performed to track compositional alterations of cell types in gene expression data. To deconvolute cell types, the immunedeconv R package (v2.0.4) with method CIBERSORT absolute was used. 

## 3. Results

The characteristics of the 60 enrolled patients hospitalized due to SARS-CoV-2 infection are shown in Table 1. 

The mean age was 63 years (SD 14.8), 23 (38.3%) were women, 11 (18.3%) had diabetes mellitus, 14 (23.3%) had dyslipidemia, 5 (8.3%) had chronic heart disease, and 5 (8.3%) had chronic pulmonary diseases. The median time from symptom onset until hospital admission was 7.8 days (SD 3.6). Most patients presented with fever (88.3%), cough (71.7%), dyspnea (45%), and diarrhea (18.3%). Almost all patients (91.7%) had pneumonia at admission, most of them bilateral (76.6%). Since many of the patients were included during the first wave, treatments included hydroxychloroquine (66.7%), lopinavir-ritonavir (28.3%), corticosteroids (53.3%), remdesivir (25%), and tocilizumab (21.7%) during hospitalization. A total of 19 (31.6%) patients required the use of a non-rebreather mask ≥24 h, 12 (20%) required a high flow nasal cannula or non-invasive mechanical ventilation, 6 (10%) were admitted to the ICU, and 3 (5%) underwent invasive mechanical ventilation. The median length of hospital stay was 9.6 days (SD 2.1). In-hospital mortality was 8.3%. A total of 9 patients (15%) met the amplified definition of ARDS at admission and 19 (31.6%) at any given time during hospitalization. Patients’ respiratory status during each day of hospitalization is represented in Figure 1. 

**Figure 2:**
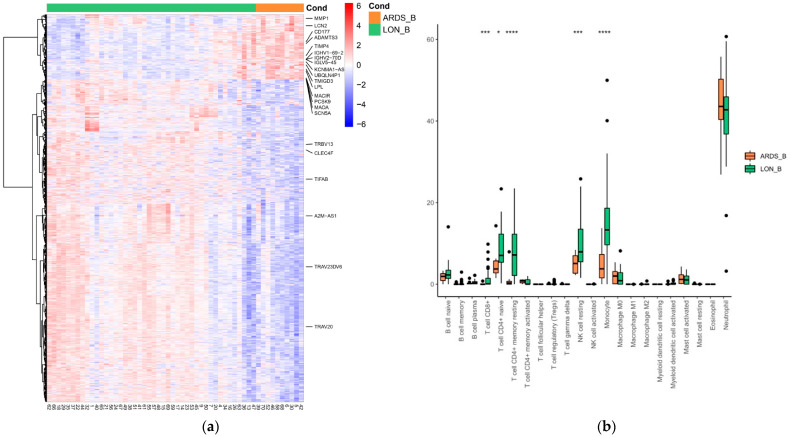
(**a**) Heatmap depicting the gene expression of differentially expressed genes (DEGs) obtained from the baseline comparison between COVID-19 patients with ARDS (ARDS_B) and those with low oxygen needs (LON_B). Each column in the figure represents a sample, and each row represents a gene. The colors in the graph indicate the magnitude of gene expression in the sample. Red indicates that the gene is highly expressed in the sample, and blue indicates that the gene expression is low. Genes included have an absolute log2 fold change of more than 1 and an adjusted *p*-value of <0.05. Genes involved in immunoglobulin production (e.g., IGHV1-69-2, IGHV2-70D, TMIGD3, IGLV5-45), monocytes/macrophages (e.g., MAOA, MACIR), neutrophil activation (e.g., CD177, LCN2), and NF-Kappa B activation were upregulated in ARDS patients, while genes associated with T-cell activation were downregulated. (**b**) Baseline comparison between patients with ARDS (ARDS_B) and those with low oxygen needs (LON_B) using CIBERSORT deconvolution analysis and comparison. Significance is noted by: * for *p*-value < 0.05, *** for *p*-value < 0.001 and **** for *p*-value < 0.0001.

Baseline comparison (within 24 h of admission) of patients with ARDS with those with low oxygen needs (maximum 6 L/min, *n* = 44) showed 2572 genes with log2-fold changes above 1. Several genes associated with T-cell activation (e.g., TRAV20, TRBV13, TRAV23DV6) and carbohydrate and galactose (e.g., CLEC4F) binding were found to be downregulated in ARDS. In contrast, many upregulated genes identified in ARDS are involved in immunoglobulin production (e.g., IGHV1-69-2, IGHV2-70D, TMIGD3, IGLV5-45), monocytes/macrophages (e.g., MAOA, MACIR), neutrophil activation (e.g., CD177, LCN2), and NF-Kappa B activation (e.g., upregulation of PCSK9 and downregulation of TIFAB). In agreement with these results, CIBERSORT deconvolution analysis showed a decrease in the population of naive CD4^+^ T cells, resting memory CD4^+^ T cells, CD8^+^ T cells, resting NK cells, and monocytes in samples from patients with ARDS. The opposite pattern was observed in the neutrophil cell population. Various upregulated genes identified in ARDS control lipid metabolic functions (e.g., OLAH, PCSK9, ACBD7, LPL, FABP2), polyubiquitination (e.g., SCN5A, UBQLN4P1, GRB10), and metalloproteinases (e.g., ADAMTS3, TIMP4, MMP1, MMP8). Interestingly, a variety of long non-coding RNAs (e.g., KCNMA1-AS1, AL592158.1, AC012146.1, IRAIN, A2M-AS1, PVT1, etc.), many of them probably involved in epigenetic control, were differentially expressed in patients with ARDS. In addition, we found significant increased and decreased levels of several non-coding microRNAs (miRNAs). Pathway analysis showed significantly enriched IL-6 and JAK-STAT3 signaling in ARDS. On the other hand, two specific enriched pathways, related to Myc V2 targets and WNT/β-catenin signaling, were identified in patients with less severe pneumonia. Figure 2 shows the heatmap, and the comparison of the CIBERSORT distribution at baseline of patients with ARDS to those with low oxygen. 

**Figure 3:**
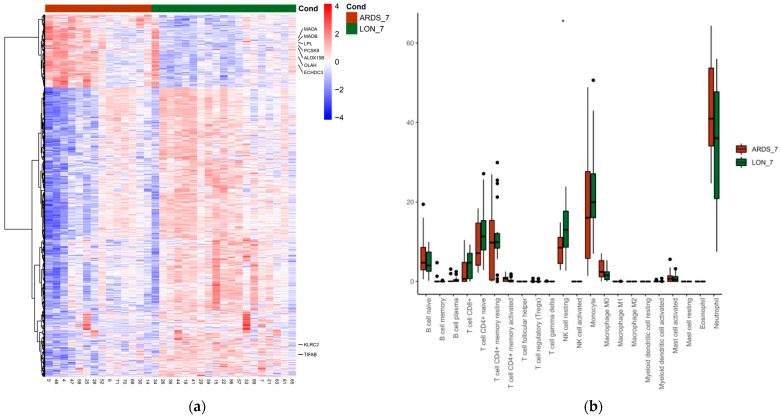
(**a**) Heatmap depicting the gene expression of differentially expressed genes (DEGs) comparing COVID-19 patients with ARDS at any given time by day 7 of hospital admission (ARDS_7) and those who did not (LON_7). Each column in the figure represents a sample, and each row represents a gene. The colors in the graph indicate the magnitude of gene expression in the sample. Red indicates that the gene is highly expressed in the sample, and blue indicates that the gene expression is low. Genes included have an absolute log2 fold change of more than 1 and an adjusted *p* value of <0.05. Genes related to lipid control (e.g., OLAH, ECHDC3, PCSK9, LPL), oxidation (e.g., MAOA, MAOB), polyubiquitination, and metalloproteinases were upregulated in patients who had presented with ARDS by day 7. (**b**) COVID-19 patients with ARDS at any given time by day 7 of hospital admission (ARDS_7) and those who did not (LON_7) CIBERSORT deconvolution analysis and comparison. Significance is noted by: * for *p*-value < 0.05.

When comparing whole blood transcriptomics at day 7 in all patients who had developed ARDS at any time with those without, 1149 significant differentially expressed genes were found. Figure 3 shows the heatmap and CIBERSORT distribution at day 7.

**Figure 4:**
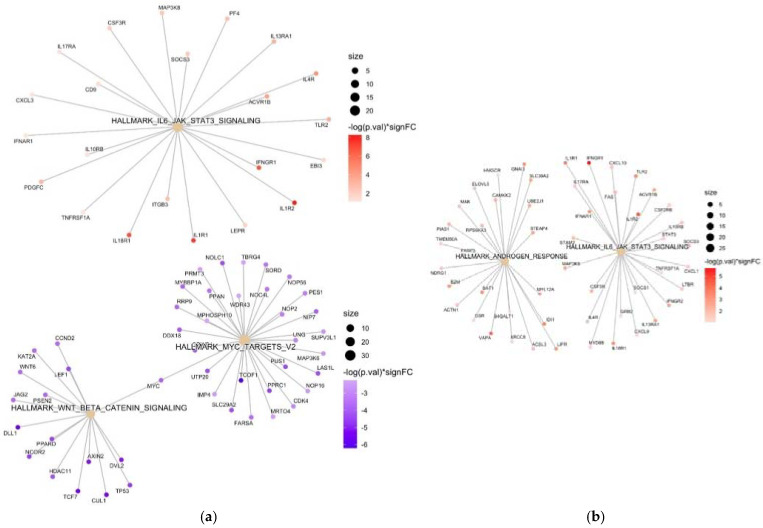
(**a**) Network plot of enriched terms at baseline comparing COVID-19 patients with and without ARDS. Target genes of each of the pathways are shown in colored circles; enriched (upregulated) genes in ARDS patients are represented in red, while downregulated genes are represented in blue, with the color intensity corresponding to increasing statistical significance. Results showed enriched IL-6 and JAK STAT3 signaling in ARDS patients, while pathways related to Myc V2 targets and WNT/β-catenin signaling were downregulated. (**b**) A network plot of enriched terms comparing COVID-19 patients with ARDS at any given time by day 7 of hospital admission to those without showed enhanced expression of IL-6, JAK-STAT3 signaling, and genes related to androgen response in ARDS patients.

We found an upregulation of genes related to lipid control (e.g., OLAH, LPL, ECHDC3, ALOX15B, PCSK9), oxidation (e.g., MAOA, MAOB), polyubiquitination, and metalloproteinases in patients with ARDS by day 7 of hospitalization. Conversely, TIFAB (which enhances NF-kappa B inhibition) and KLRC2 (involved in NK activation) were downregulated in patients with ARDS. Again, we found significant increased and decreased levels of several lncRNAs and miRNAs between patients with ARDS at any given time and those without at day 7. Pathway analysis found a significantly enhanced expression of IL-6 and JAK STAT3 signaling and genes related to androgen response in patients with ARDS compared to those with those with lower oxygen needs. A pathway analysis comparing baseline and day 7 is shown in Figure 4.

## 4. Discussion

The host-pathogen interaction in COVID-19 is complex and leads to heterogeneous clinical presentations. This means that there is a particular interest in understanding the underlying transcriptomic host response to SARS-CoV-2 infection. In this study, by stratifying patients according to oxygen requirement, we attempted to reduce the heterogeneity in the transcriptomic profiles observed in previous studies in hospitalized COVID-19 patients [5,21,22]. 

Our study adds to the evidence that a dysregulated inflammatory response [5,25,26] is the major driver behind severe pneumonia in SARS-CoV-2 infection. Patients with ARDS at baseline showed an upregulation of genes related to IL-6 and JAK-STAT3 signaling and neutrophil activation, as seen in other studies [5,17,19], a downregulation of T-cell activation, and a subsequent loss of CD4^+^ T cells [5,20]. We also observed an increased expression of genes related to reactive oxygen species metabolism at baseline in ARDS, an increase that a previous study had reported at later stages of COVID-19 [5]. This discrepancy is likely explained by a delay in hospital admission in our cohort since many of the patients presented to the emergency department with already established ARDS. Our findings concur with other transcriptomic studies that have encountered an upregulation of genes related to protein polyubiquitination [19] and metalloproteinases [27] in later stages of COVID-19 induced ARDS. On the other hand, in non-ARDS COVID-19 patients, we observed an increased expression of Wnt/β-catenin signaling and Myc V2 targets, a subgroup of genes regulated by Myc. Wnt/β-catenin pathway components modulate T-cell priming and infiltration [28] and negatively regulate NF-κB [29], thus enhancing viral tolerance and limiting inflammation. Myc, in addition to its well-known role in cancer, directly programs immune suppression by inhibiting macrophage activation [30] and preventing endothelial inflammation [31]. 

Our results further highlight the importance of long non-coding RNAs [32] and microRNAs [33] as emerging regulators in SARS-CoV-2 infection. Patients with ARDS at baseline presented higher levels of CLRN1-AS1, a lncRNA that inactivates the Wnt/β-catenin signaling pathway [34], and IRAIN, which enhances the formation of an intrachromosomal promoter loop of IGF1R [35]. Higher serum levels of IGF1R correlate with COVID-19 mortality [36]. On the other hand, the expression of the lncRNAs A2M-AS1, LEF-AS1, and RORA-AS-1 was significantly decreased in patients with ARDS. A2M-AS1 probably has an anti-proliferation and pro-apoptosis effect [37], and LEF1-AS1 and RORA-AS-1 have been found to be involved in T cell differentiation in COVID-19 patients [38]. The decreased levels of A2M-AS-1 in severe COVID-19 patients are in accordance with a previous study [39]. 

Our study has several limitations that should be acknowledged. Firstly, the majority of patients correspond to the first wave, in which lineage A predominated [40]. Subsequent SARS-CoV-2 variants and subvariants might have elicited different host responses. Secondly, the sample size was relatively small—only 60 patients, of whom only 19 developed ARDS. However, one of the strengths of the study is that the patients were followed up every day, which allowed an accurate assessment of their respiratory status. Additionally, our results are validated by the concordance of the cell composition of the samples studied with previous studies performed on single-cell RNA sequencing [41,42,43,44]. 

In conclusion, we found a dysregulated inflammatory response in COVID-19 ARDS patients with an increased expression of genes related to pro-inflammatory molecules and neutrophil and macrophage activation at admission, in addition to the loss of immune regulation. This led to a higher expression of genes related to reactive oxygen species, protein polyubiquitination, and metalloproteinases. These results should now be assessed in new studies with other variants (Omicron subvariants) and in populations with preexisting immunity.

## Figures and Tables

**Table 1:** Patients’ characteristics.

Patients’ Characteristics	*n* (%)
Age (mean, SD)	63 (14.8)
Woman	23 (38.3%)
Active tobacco use	0 (0%)
Diabetes mellitus	11 (18.3%)
Dyslipidemia	14 (23.3%)
Preexisting pulmonary diseases	5 (8.3%)
Heart disease	5 (8.3%)
Stroke	3 (5%)
Renal failure	2 (3.3)
Dementia	2 (3.3%)
Solid organ transplant recipient	2 (3.3)
Obesity (BMI > 30)	31 (51.7%)
Morbid obesity (BMI > 40)	4 (6.7%)
Clinical presentation	
Duration of symptoms (mean days, SD)	7.8 (3.6)
Fever	53 (88.3%)
Cough	43 (71.7%)
Dyspnea	27 (45%)
Diarrhea	11 (18.3%)
Cephalea	8 (13.3%)
Altered consciousness	5 (8.3%)
Mean room air saturation (%, SD)	94.9% (4)
Room air pulsioximetry <94% (%)	26 (43.3%)
Mean respiratory rate (SD)	24.2 (6.9)
Respiratory rate >30	11 (18.3%)
Mean lymphocytes (×10^6^, SD)	1083 (465)
Mean C reactive protein (mg/L, SD)	128 (107)
Pneumonia at presentation	55 (91.7%)
Bilateral pneumonia at presentation	46 (76.6%)
COVID-19 treatment	
Lopinavir-ritonavir	17 (28.3%)
Hydroxychloroquine	40 (66.7%)
Remdesivir	15 (25%)
Tocilizumab	13 (21.7%)
Steroids	32 (53.3%)
Outcomes	
Use of non-rebreather mask ≥24 h any given time	19 (31.6%)
Use of high flow nasal cannula or non-invasive mechanical ventilation any given time	12 (20%)
ICU admission	6 (10%)
Median APACHE II score at ICU admission (SD)	12.33 (2.7)
Mechanical ventilation	3 (5%)
Nosocomial infection	8 (13.3%)
Median length of hospitalization stay (days, SD)	9.6 (2.1)
In-hospital mortality	5 (8.3%)

## Data Availability

The gene expression data set for this study will soon be made public in the Gene Expression Omnibus [36].

## Supplementary Materials

The following supporting information can be downloaded at: https://www.mdpi.com/article/10.3390/biomedicines11051348/s1, Case report form, IMIM’s biosecurity measures.
