# Supplementary material for: Dynamics of Gene Expression Profiling and Identification of High-Risk Patients for Severe COVID-19
Source: Biomedicines. 2023 May 3;11(5):1348. doi: 10.3390/biomedicines11051348 (PMC10216228; doi:10.3390/biomedicines11051348)
Supplement: Supplementary file 1 [file biomedicines-11-01348-s001.zip › biomedicines-2353623-supplementary.pdf]

Supplementary Material

<u>Case report form</u>:

Patient identification number:____
Date of informed consent signing: _____
Date of COVID-19 diagnosis (defined as first microbiological test):______
Positive SARS-CoV-2 test: Antigen/PCR
Nosocomial adquisition (defined as a negative PCR on admission and compatible symptom with COVID-19 with the development of COVID-19 and a positive PCR test >5 days of admission): Y/N
Flu vaccination (within the same season): Y/N
Date of admission: ____
Year of birth:____
Sex at birth: W/M
Pregnancy: Y/N
Risk factors (whenever the patients present any of the following comorbidities): Y/N

- -Chronic lung disease:
  - COPD: Y/N
  - Asthma: Y/N
  - SAHS: Y/N
  - Other:___
- -Chronic heart disease:
  - Ischemic heart disease (defined as proven myocardial necrosis or previous requirement of coronary angioplasty or stent placement): Y/N
  - Chronic heart failure (defined as having presented at least one previously proven episode of acute heart failure with elevated pro-BNP): Y/N
  - Arrhythmia (defined as having at least one previous episode of atrial fibrillation, atrial flutter or ventricular tachycardia): Y/N
- -Stroke: Y/N
- -Hemiplegia: Y/N
- -Peripheral vasculopathy (defined as need for carotid stent placement, arterial claudication, peripheral vascular bypass): Y/N
- -Chronic renal failure: mild (creatinine between 1.5 and 3mg/dL), moderate-severe (>3mg/dl or dyalisis)
- -Hepatopathy: mild (defined as chronic viral hepatitis or cirrhosis without portal hypertension), moderate-severe (defined as cirrhosis with portal hypertension).
- -HIV infection: Y/N
  - AIDS: Y/N
  - Antiviral treatment: Y/N
  - Latest CD4+ count and % of total lymphocytes: _____/______
- -Connective tissue disease (defined as any autoimmune rheumatological condition needing active treatment): Y/N
- -Acute leukemia: Y/N
- -Any other hematological disease (chronic myelodysplastic and myeloproliferative syndrome): Y/N
- -Dementia: Y/N

-Obesity (defined as BMI>30): Y/N
-Morbid obesity (defined as BMI >35): Y/N
-Resident of a care centre: Y/N

-Active smokers: Y/N
-Active alcohol consumption (defined as an intake higher than 14 units per week): Y/N
-Antibiotic within the last 3 months: Y/N Name of antibiotics: _______
-Antibiotic for this episode: Y/N Name of antibiotic:_____
-Chronic statin use: Y/N
-Symptoms duration (in days): ____
-Fever: Y/N Days of fever:____ Chills: Y/N
-Odynophagia: Y/N
-Cough: Y/N
-Dyspnea: Y/N
-Chest pain: Y/N
-diarrhea (defined as liquid stools): Y/N
-Vomits: Y/N
-Headache: Y/N
-Decreased consciousness: Y/N
-Shock within the first 24h of admission (defined as the need for vasoactive drugs): Y/N
-Body temperature on admission:___
-Arterial blood pressure on admission (systolic and diastolic):____
-Oxygen saturation on room air (if receiving oxygen supplementation on admission, the oxygen saturation on room air recorded by the first responders): ___
-Heart rate on admission:____
-Respiratory rate on admission:____
-Lung auscultation (day of basal sampling): Normal/crackles/wheezes
-White blood cells on admission (10^9/L): ___
-Total neutrophils on admission (10^9/L): ____
-Total lymphocytes on admission (10^9/L):____
-Hemoglobin on admission (g/dl):___
-Platelets on admission (10^9/L):____
-Sodium on admission (mmol/L):___
-Potassium on admission (mmol/L):___
-Glucose on admission (mg/dL):____
-Urea on admission (mg/dL):____
-C-reactive protein on admission (g/L): ____
-pH on admission:___ PaCo2 on admission:___ Bicarbonate on admission:___ PaO2 on admission:____ Oxygen flow:___
-Chest X-ray: Normal/Unilateral interstitial lung infiltrates/Bilateral interstitial lung infiltrates/Unilateral alveolar lung infiltrate/Bilateral alveolar lung infiltrate
+ Pneumonia (defined as new pulmonary infiltrates): Y/N
+Pleural effusion: Y/N

-Sputum culture: Not performed/negative/positive Identified microorganism: ____
-Blood cultures: Not performed/negative/positive Identified microorganism: ____
-Pleural liquid culture: Not performed/negative/positive Identified microorganism: ____
-Bronchoscopy (during hospitalization): Not performed/negative/positive Identified microorganism:____
-Pneumococcal urinary antigen: Not performed/negative/positive
-Legionella urinary antigen: Not performed/negative/positive

-Antiviral treatment: Y/N
Lopinavir/ritonavir: Y/N Start date: ____
Hydroxycloroquine: Y/N Start date:____
Azithromycin: Y/N Start date:____
Remdesivir: Y/N Start date:____
-Immunomodulatory treatment: Y/N
Tocilizumab: Y/N
Immunoglobulins: Y/N
Hyperimmune plasma: Y/N
-Corticosteroids: Y/N Start date:____ End date: _____ Type steroids: ______
Maximum daily dose of corticosteroid (prednisone equivalent):_____
-Empirical antibiotic treatment Y/N Antibiotic:____________
-Complications: Y/N
ICU admission: Y/N Date of ICU admission: ____ Apache-II score on admission: __
Date of ICU discharge:_____
Respiratory complications: Y/N
Use of non-rebreather mask (NRB; Fio2 >80%) for >24h: Y/N
Non-invasive mechanical ventilation (NIV) or high-flow nasal cannula (HFNC) for >24h: Y/N
Invasive mechanical ventilation for >24h: Y/N
Amplified definition of ARDS: Y/N
New pleural effusion: Y/N
Empyema: Y/N
Cardiac complications: Y/N
Type of cardiac complication: Coronary syndrome/heart failure/new onset trial fibrillation or flutter/paroxysmal atrial fibrillation or flutter/chronic trial fibrillation or flutter with rapid ventricular response/Ventricular tachycardia of fibrillation.
Date of cardiac complication: ____
Stroke: Y/N
Acute renal failure (defined as having injury or failure according to the *RIFLE criteria*; at least a two-fold increase in serum creatinine, or 5a 0% fall in GFR, or urine output <0.5 mL/kg per hour for 12 hours): Y/N
Shock during hospitalization (defined as the need for vasoactive drugs to maintain adequate tissue perfusion): Y/N
Nosocomial infection: Y/N
Hepatotoxicity (defined as ALT or AST >x5ULN): Y/N
Other complication:_____
Death during hospitalization: ____
Date of discharge or death:____
Readmission within 30 days: _____

Date of basal sample: ____
Respiratory status: Respiratory rate:___ Peripheral saturation: ___ Supplemental oxygen: Y/N -> FiO2:___ Oxygen flow:___ use of NRB: Y/N HFNC: Y/N NIV: Y/N MV: Y/N
Date of sample day 3: ___
Respiratory status: Respiratory rate:___ Peripheral saturation: ___ Supplemental

oxygen: Y/N -> FiO2:___ Oxygen flow:___ use of NRB: Y/N HFNC: Y/N NIV: Y/N MV: Y/N

Date of sample day 7: ___

Respiratory status: Respiratory rate:___ Peripheral saturation: ___ Supplemental oxygen: Y/N -> FiO2:___ Oxygen flow:___ use of NRB: Y/N HFNC: Y/N NIV: Y/N MV: Y/N

<u>IMIM's biosecurity measures</u>:

The COVID room has an antechamber where the technicians changed their personal protection equipment including gloves, robe, and facemask. Inside the room all the samples were processed by staff wearing double gloves, FFP3 facemask, plastic robe, cap, and plastic shoe covers. Furthermore, the samples were processed inside a biosecure laminar flow cabin with HEPA filter and following the biosafety COVID guidelines developed by the IMIM.
